# Moving from lipids to leukocytes: inflammation and immune cells in atherosclerosis

**DOI:** 10.3389/fcell.2024.1446758

**Published:** 2024-08-05

**Authors:** Maxim E. Annink, Jordan M. Kraaijenhof, Erik S. G. Stroes, Jeffrey Kroon

**Affiliations:** ^1^ Department of Vascular Medicine, Amsterdam Cardiovascular Sciences, Amsterdam University Medical Center, University of Amsterdam, Amsterdam, Netherlands; ^2^ Department of Experimental Vascular Medicine, Amsterdam Cardiovascular Sciences, Amsterdam University Medical Center, University of Amsterdam, Amsterdam, Netherlands; ^3^ Laboratory of Angiogenesis and Vascular Metabolism, VIB-KU Leuven Center for Cancer Biology, Leuven, Belgium; ^4^ Laboratory of Angiogenesis and Vascular Metabolism, Department of Oncology, KU Leuven and Leuven Cancer Institute (LKI), Leuven, Belgium; ^5^ Amsterdam Cardiovascular Sciences, Atherosclerosis and Ischemic Syndromes, Amsterdam, Netherlands

**Keywords:** vascular medicine, atherosclerosis, inflammation, cardiovascular disease, immune cells

## Abstract

Atherosclerotic cardiovascular disease (ASCVD) is the most important cause of morbidity and mortality worldwide. While it is traditionally attributed to lipid accumulation in the vascular endothelium, recent research has shown that plaque inflammation is an important additional driver of atherogenesis. Though clinical outcome trials utilizing anti-inflammatory agents have proven promising in terms of reducing ASCVD risk, it is imperative to identify novel actionable targets that are more specific to atherosclerosis to mitigate adverse effects associated with systemic immune suppression. To that end, this review explores the contributions of various immune cells from the innate and adaptive immune system in promoting and mitigating atherosclerosis by integrating findings from experimental studies, high-throughput multi-omics technologies, and epidemiological research.

## Graphical Abstract

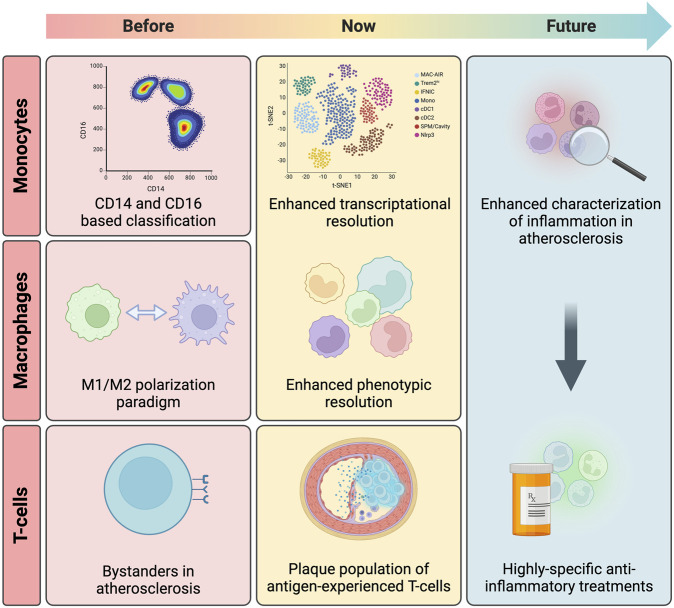

## 1 Introduction

In recent years, atherosclerotic cardiovascular disease (ASCVD) has grown to be the leading cause of mortality worldwide (Vos et al., 2020). The formation of atherosclerotic plaques, or atherogenesis, is preceded by increased vessel wall activation and increased vascular permeability. Following this, in a process that spans many years, accumulation of low-density lipoprotein (LDL) within the vascular endothelium eventually leads to plaque formation. This process occurs mainly at sites of the vasculature that are characterized by disturbed blood flow, such as branch points and bifurcations. As the plaque grows, sudden occlusion can follow rupture or erosion of the plaque’s surface, leading to ischemic events with clinical consequences such as myocardial infarction and stroke (Farb et al., 1996; Libby et al., 2011; Bentzon et al., 2014).

Experiments in human and murine models, including the well-known *Apoe* ^−/−^, apoE*3-Leiden.CETP, and *Ldlr* ^−/−^ mice models, have countered the traditional view that atherosclerosis is a disease of mere passive lipid accumulation. On the contrary, it is now widely accepted that low-grade inflammation is a hallmark of the pathophysiology of atherosclerosis. Leukocytes densely populate the arterial walls both in healthy and affected individuals. Their number and composition, however, differ in health and disease. Plaque inflammation is driven by involvement of both the innate and adaptive immune system, which is caused by persistent pro-inflammatory triggers that facilitate both plaque progression and the occurrence of plaque rupture and erosion.

Clinicians have traditionally focused on vigorously lowering plasma cholesterol levels as the predominant approach to stalling plaque development and prevention of cardiovascular events. This paradigm, however, is due for reconsideration. In a considerable proportion of patients that receive optimal lipid-lowering therapy in accordance with current guidelines, a residual inflammatory risk of recurrent cardiovascular complications remains (Sampson et al., 2012). A recent meta-analysis of clinical trials, encompassing over 30.000 patients with a history of ASCVD, showed that residual inflammatory risk, defined as high-sensitivity C-reactive protein ≥2 mg/L, is a larger driver of recurrent cardiovascular events than risk attributed to residual LDL cholesterol (LDL-C) in patients already receiving optimal lipid lowering therapy (Ridker et al., 2023). The Canakinumab Anti-inflammatory Thrombosis Outcomes Study (CANTOS) was the first landmark study to demonstrate that a monoclonal antibody (canakinumab) targeting IL-1β could significantly reduce the recurrence rates of cardiovascular events independent of changes at the lipid level (Ridker et al., 2017). Similar results were obtained using low doses of the anti-inflammatory drug colchicine (Nidorf et al., 2020). It is therefore now widely recognized that mitigating inflammation is of paramount importance to further reduce residual cardiovascular risk. It should be noted however, that trials like CANTOS have shown that broad targeting of inflammation comes with serious adverse side effects. Additionally, in the Cardiovascular Inflammation Reduction Trial (CIRT), administration of a low dose of the broad anti-inflammatory agent methotrexate did not result in a reduction of cardiovascular events, which is indicative of the complexity of inflammatory pathways in atherosclerosis. Research efforts should therefore focus on more specific anti-inflammatory therapies that target, for instance, one particular immune cell type or inflammatory process that is causative for atherosclerosis. They should also recognize that inflammation is a crucial component of host homeostasis (Xu et al., 2022). Effective therapeutic strategies might therefore involve not just the suppression of “bad” inflammation but also the enhancement of “good” inflammation to maintain a balanced immune response in the face of pro-atherosclerotic triggers. In light of this, detailed knowledge about the inflammatory processes governing atherogenesis is indispensable. The introduction of novel high-throughput methodologies, such as single-cell RNA sequencing (scRNA-seq) has greatly accelerated our ability to characterize the immune landscape in atherosclerosis. These techniques will continue to enhance our understanding of the complex cellular interactions and molecular pathways driving disease progression, paving the way for novel personalized and effective anti-inflammatory therapies that are cell- or pathway-specific.

In this review, we will explore the functions of various immune cells and the molecular mechanisms at play in atherosclerosis, emphasizing contributions from high-throughput technologies where relevant. By using these insights to find possible therapeutic targets, it paves the way for novel methods to mitigate inflammatory cardiovascular risk.

## 2 Endothelial cell activation primes the vessel wall for an inflammatory response

### 2.1 Laminar shear stress induces an anti-inflammatory and atheroprotective effect

**FIGURE 1:**
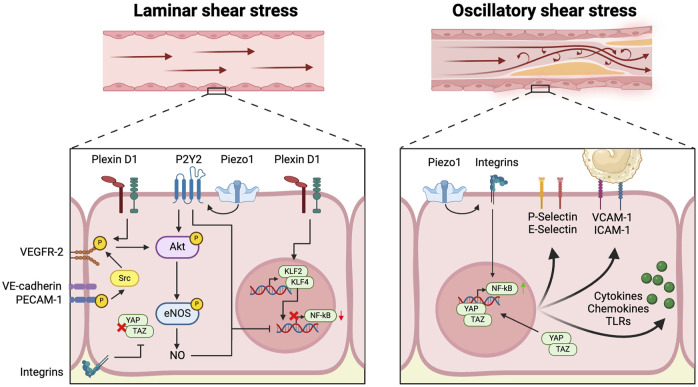
Mechanosensing of disturbed flow induces endothelial activation At the apical surface of the vascular endothelial cell, Plexin D1, Piezo1 and P2Y2 act as mechanosensors and sense laminar flow, leading to activation of Akt and endothelial nitric oxide synthase (eNOS), in addition to upregulation of Kruppel-like transcription factors (KLF) 2 and 4 and downregulation of the pro-inflammatory nuclear factor-κB (NF-κB). At cell-cell junctions, vascular endothelial cadherin (VE-cadherin) works in concert with platelet endothelial cell adhesion molecule (PECAM1) and vascular endothelial growth factor receptor 2 (VEGFR2) to induce Akt activation in response to laminar flow. At the basal membrane, integrin signaling downregulates yes-associated protein (YAP) and transcriptional coactivator with PDZ-binding motif (TAZ) signaling. In contrast, Piezo1 induces expression of NF-κB through integrin signaling in response to disturbed flow. Coupled with unbridled YAP-TAZ signaling, this leads to a variety of pro-inflammatory responses: upregulation of P-Selectin, E-selectin, Vascular Cell Adhesion Molecule-1 (VCAM-1) and Intercellular Adhesion Molecule-1 (ICAM-1), and increased production of various cytokines, chemokines and Toll-like receptors (TLRs).

The arterial wall is lined by a single layer of endothelial cells (EC), which are constantly exposed to variations in shear stress patterns and regulated by a multitude of mechanical and molecular factors that either promote or mitigate inflammation (Luscinskas Francis and Gimbrone, 1996). EC activation serves as a first line of defense against atherogenic stressors and potentiates an increase in interactions between ECs and circulating immune cells. It is largely driven by variations in blood flow-induced shear stress throughout the arterial vasculature (Gimbrone et al., 2000). High laminar shear stress (LSS), occurring in regions characterized by stable flow triggers various downstream anti-inflammatory signals within ECs. These signals are sensed by a complex network of mechanosensitive protein complexes present on cell-cell junctions and the apical and basal endothelial surface of ECs (Figure 1) (Demos et al., 2020).

On the apical surface, the cation channel Piezo1 relays downstream signals through the activation of the P2Y2 and Gαq/11 pathways, which in turn activate Akt, upon mechanical detection of LSS (Iring et al., 2019). At cell-cell junctions, LSS leads to the phosphorylation of the mechanosensory cell-cell adhesion protein Platelet Endothelial Cell Adhesion Molecule-1 (PECAM-1). Through its interaction with Vascular Endothelial Cadherin (VE-cadherin), phosphorylated PECAM-1 initiates Src-dependent phosphorylation of VEGFR2 and -3 and subsequent Akt activation (Tzima et al., 2005; Coon et al., 2015). Recent research has shed light on the role of the guidance receptor plexin D1, which has a mechanosensing function on the apical endothelial membrane. This receptor forms a complex with Neuropilin 1 (NRP1) and Vascular Endothelial Growth Factor Receptor 2 (VEGFR2) to contribute to activation of the latter in response to LSS (Mehta et al., 2020). These mechanosensory pathways, involving Piezo1, plexin D1, and PECAM-1, converge within the endothelial cell to activate various anti-inflammatory signaling pathways (Fleming et al., 2005; Wang et al., 2015; Iring et al., 2019; Mehta et al., 2020). This triggers a series of downstream atheroprotective effects, such as the upregulation of endothelial nitric oxide synthase (eNOS), leading to increased production of endothelial nitric oxide (NO) and thus increasing endothelial barrier integrity and reducing oxidative stress (Fleming et al., 2005; Wang et al., 2015; Iring et al., 2019; Mehta et al., 2020). Even more significantly, LLS-induced mechanosignalling increases transcription of Kruppel-like transcription factors (KLF) 2 and 4, which are recognized as pivotal regulators in the flow-sensitive activation of anti-atherogenic pathways (Novodvorsky et al., 2014). Notably, NO, KLF2 and KLF4, as well as Piezo1-mediated P2Y purinoceptor 2 (P2Y2) and Gαq/11 signaling inhibit the transcription of the pro-inflammatory nuclear factor kappa B (NF-κB) (Gofman et al., 1950; Albarrán-Juárez et al., 2018). Furthermore, KLF2 directly inhibits transcription of the glycolytic enzyme 6-phosphofructo-2-kinase/fructose-2, 6-biphosphatase-3 (PFKFB3), thereby repressing glycolysis in ECs and promoting a quiescent endothelial state. Doddaballapur et al. (2015) Moreover, exposure of endothelial mechanosensory integrin β to LSS at the basal membrane results in the inhibition of yes-associated protein (YAP) and transcriptional coactivator with PDZ-binding motif (TAZ) signaling within the pro-inflammatory Hippo pathway (Wang et al., 2016).

### 2.2 Oscillatory shear stress leads to endothelial cell activation

In contrast to LSS, oscillatory shear stress (OSS), predominantly occurring in branch points and bifurcations of the vasculature incites a pro-inflammatory reaction within the endothelium (Figure 1). In this scenario, attenuation of NF-κB expression and YAP-TAZ signaling by mechanosensors Piezo1, PECAM-1, and integrins is lost (Tzima et al., 2005; Albarrán-Juárez et al., 2018). Activation of Piezo1 in response to disturbed flow further induces expression of NF-κB through integrin activation (Albarrán-Juárez et al., 2018). Increased NF-κB and YAP-TAZ signaling trigger endothelial activation, as evidenced by the upregulation of monocyte adherence molecules including Vascular Cell Adhesion Molecule-1 (VCAM-1), E-Selectin, P-selectin, and Intercellular Adhesion Molecule-1 (ICAM-1), and expression of potent pro-inflammatory mediators including Toll-like receptor (TLR) 2, chemokine (C-C motif) ligand 2 (CCL2), Interleukin (IL)-6, and IL-8 (Tzima et al., 2005; Wang et al., 2016; Bondareva et al., 2019). Substantiating these experimental observations, a recent scRNA-seq study on ECs from human coronary arteries in transplanted human hearts provided compelling *ex vivo* evidence that endothelial activation is essential for atherosclerotic plaque formation. In this study, a distinct EC subpopulation that was characterized by upregulation of genes associated with inflammation and endothelial activation was identified, constituting over 80% of all ECs (Hu et al., 2021). This subpopulation was more abundantly present in atherosclerotic arteries compared to healthy controls. A similar EC phenotype was identified in a subsequent scRNA-seq study of human carotid plaques, where a large majority of intra-plaque ECs showed expression of genes such as *PECAM1* and *VCAM1* that are associated with endothelial activation (Depuydt et al., 2020).

## 3 The role of the innate immune system in atherosclerosis

### 3.1 Monocyte subtypes play different roles in atherogenesis

Monocytes are bone marrow derived immune cells capable of differentiating into macrophages and, under certain inflammatory conditions, into monocyte-derived dendritic cells (DCs). As (activated) monocytes predominantly interact with activated endothelium, they have historically played a central role in atherosclerosis research (Kim et al., 2020). Classical cardiovascular risk factors, such as dyslipidemia, lead to monocytosis through upregulation of bone marrow activity in mice (Moore et al., 2013). In humans, a positive history of ASCVD has been linked to increased metabolic activity of hematopoietic tissues of the spleen and bone marrow as well as an enhanced functional status of hematopoietic stem and progenitor cells, indicating increased hematopoietic activity as a potential driver of monocytosis and low-grade inflammation in ASCVD (van der Valk et al., 2016). The relation between cardiovascular health, bone marrow activity and atherogenesis was further studied in the Progression of Early Subclinical Atherosclerosis (PESA) study (Devesa et al., 2022). Here, classical risk factors such as metabolic syndrome, hypertension, dyslipidemia, diabetes and BMI correlated significantly with bone marrow activation on ^18^F-FDG PET/MRI. Consequently, these subjects showed increased leukocyte counts and elevated markers of inflammation, indicating low-grade systemic inflammation. In turn, bone marrow activation was associated with arterial uptake of ^18^F-FDG, indicating early plaque formation.

Within atherosclerotic plaques, monocytes transition into macrophages, which have an affinity for the uptake of modified LDL (Zernecke et al., 2020). Flow cytometry and fluorescence-activated cell sorting (FACS) are often used to classify monocyte subsets in humans based on CD14 and CD16 receptor levels: classical monocytes (∼90% of circulating monocytes; CD14^++^CD16^−^), followed by intermediate (∼5%; CD14^++^CD16^+^) and non-classical monocytes (∼5%; CD14^+^CD16^++^). A similar classification is used in mice, where Ly6^high^ monocytes correspond to human classical monocytes, and Ly6^low^ monocytes to non-classical monocytes (Mehta and Reilly, 2012). Generally, non-classical monocytes are ascribed a role in homeostasis and atheroprotection, as depletion of non-classical monocytes in murine models has resulted in aggravation of atherogenesis and increased apoptosis of ECs (Quintar et al., 2017). Intravital microscopy experiments have shown that classical monocytes engage in ICAM-1 and -2-dependent “patrolling” along the endothelial surface of murine atherosclerotic arteries (Quintar et al., 2017). Furthermore, they typically avoid entering the subendothelial space (Quintar et al., 2017). Classical monocytes, on the other hand, are attracted to the atherosclerosis-prone endothelium, targeted by CCL2 on the endothelial surface in a manner reliant on the leukocyte C-C Chemokine Receptor Type 2 (CCR2). Their important role in atherogenesis is confirmed in murine knock-out models of CCR2 and CCL2*,* which show a significant reduction in atherosclerosis formation compared to wild type mice (Boring et al., 1998; Gu et al., 1998). Aside from CCL2, CCL5 plays a role in chemotaxis of classical monocytes through interaction with leukocyte CCR5 in the atherosclerotic vessel wall. In murine atherosclerosis models, CCL5 expression is significantly enhanced in the vessel wall compared to wildtype mice. CCR5 expression is upregulated in tandem in circulating monocytes. Various separate experiments involving administration of function-blocking antibodies to CCR5, genetic depletion of CCR5, and genetic depletion of CCL2 have all demonstrated a significant reduction in lesion size (Tacke et al., 2007; Combadiè et al., 2008; Jongstra-Bilen et al., 2021). Finally, it is this specific group of classical monocytes that undergoes expansion in reaction to hypercholesterolemia and atherosclerosis, whereas formation of non-classical monocytes is impaired under these circumstances (Swirski et al., 2007). This finding underscores the largely opposite roles of the two types of monocytes. Nonetheless, the precise role of non-classical monocytes in atherosclerotic plaque formation and inflammation in humans remains subject of further study.

Although monocyte subtypes have traditionally been classified based on CD14 and CD16 surface expression, recent advancements in scRNA-seq and mass cytometry have challenged this triadic categorization, uncovering more monocyte diversity than initially thought. For example, a study using scRNA-seq in human monocytes found that monocytes that had previously been defined as intermediate subtype showed considerable overlap with the classical and nonclassical subtypes. Surprisingly, these monocytes clustered into two additional and previously undefined clusters as well, introducing novel heterogeneity of intermediate monocytes (Villani et al., 2017). Subsequent investigations in a study using machine learning to reclassify monocyte subpopulations based on scRNA-seq and mass cytometry data, proposed that one of these novel monocyte subtypes represented a cluster of Natural Killer (NK) cells (Dutertre et al., 2019). Likewise, other potential novel monocyte subtypes have been proposed following studies using a variety of different high-throughput strategies and analytical approaches, both in humans (Roussel et al., 2017; Thomas et al., 2017) and in mice (Mildner et al., 2017). Even though reaching a consensus on these subpopulations and their distinct functions will require further research, the enhanced subclassification of monocytes through high-throughput techniques and omics approaches are likely to prove essential to atherosclerosis research. In this context, it is advisable to strive for standardization of markers and clustering methods used to ensure reproducible identification of cell clusters. More precise identification of monocyte subsets will enable dissection of their specific pro-inflammatory and pro-atherosclerotic contributions allowing for more focused and hypothesis-driven research.

### 3.2 Monocytes migrate into the vessel wall and differentiate into foam cells

**FIGURE 2:**
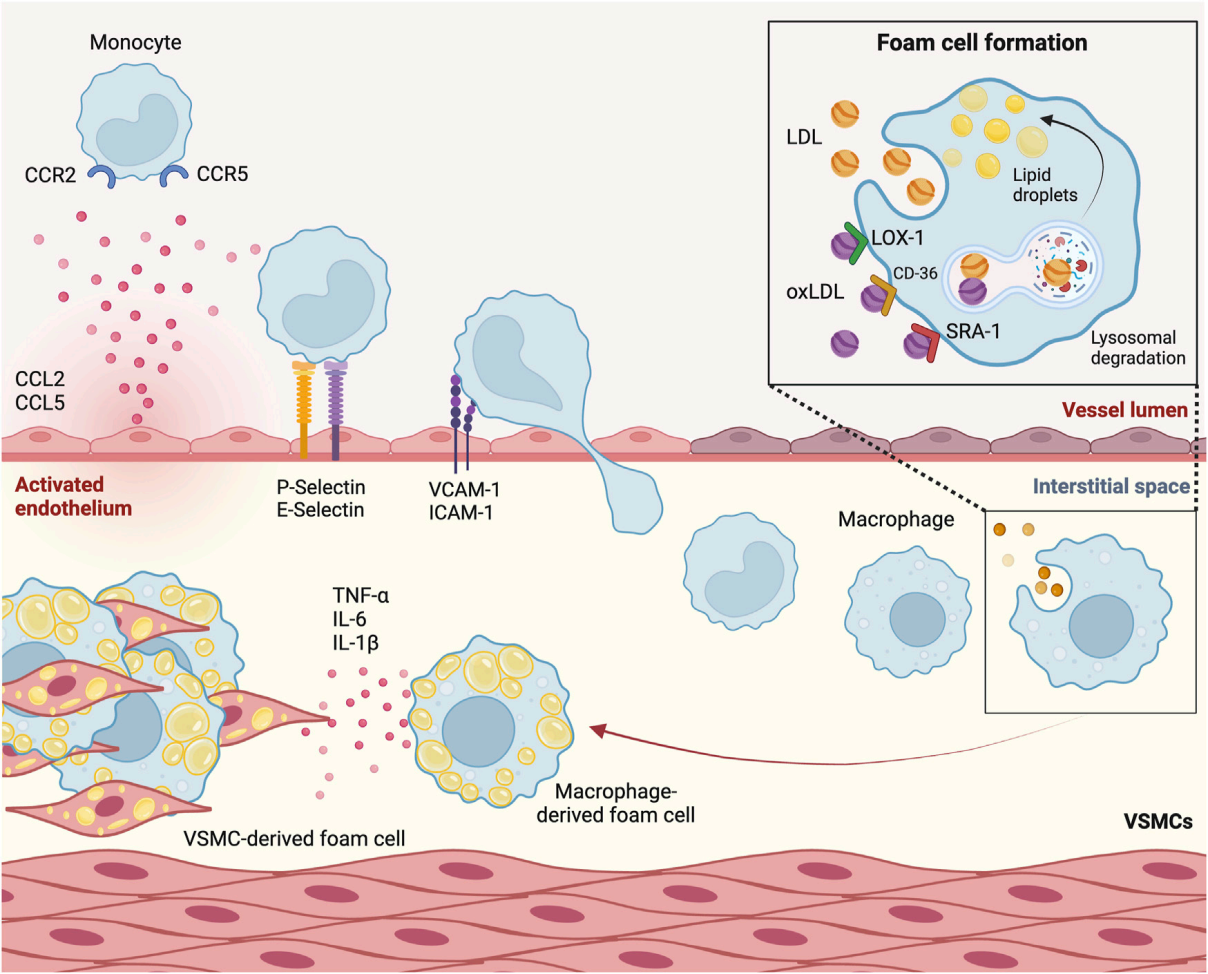
Monocytes transmigrate through the activated endothelium to form foam cells. Circulating monocytes are attracted to the activated endothelium by chemokines chemokine (C-C motif) ligand 2 (CCL2) and CCL5. P-selectin and E-selectin on the endothelial membrane are involved in monocyte recruitment. Once in close proximity, molecules such as Vascular Cell Adhesion Molecule-1 (VCAM-1) and Intercellular Adhesion Molecule-1 (ICAM-1) initiate monocyte transmigration. Once in the subendothelial space, monocytes differentiate into macrophages. Through a process of phagocytosis mediated by scavenger receptor A1 (SRA1), Lectin-like Oxidized Low-Density Lipoprotein Receptor-1 (LOX-1) and CD-36, and through micropinocytosis, macrophages take up (oxidized) low-density lipoprotein [(ox)LDL]. These lipoproteins undergo lysosomal degradation, after which their byproducts are stored as lipid droplets, leading to foam cell formation. Sustained influx of lipoproteins leads to secretion of cytokines such as tumor necrosis factor α (TNF-α), interleukin-6 (IL-6) and IL-1β. VSMC, vascular smooth muscle cell.

Once near the activated endothelium, monocytes undergo transient rolling interactions, followed by firm adhesion mediated by integrins and chemokine activation, ultimately leading to their migration across the endothelium barrier into the subendothelial space (Figure 2) (Timmerman et al., 2016). Early experiments in mice models of atherosclerosis involving genetic depletion of P-selectin made clear that this protein facilitates leukocyte rolling, extravasation and by consequence, plaque formation (Mayadas et al., 1993; Johnson et al., 1997; Dong et al., 2000). Subsequent experiments involving murine knockout models of its ligand, leukocyte P-selectin glycoprotein ligand-1 (PSGL-1), confirmed the interaction of these proteins as a major driver of monocyte recruitment in atherosclerosis (An et al., 2008). Moreover, involvement of other adhesion molecules on the activated endothelium, such as ICAM-1, ICAM-2 and VCAM-1, have been associated with progression of atherosclerosis in *Apoe* ^*−/−*^ mice following coronary ligation (Sager et al., 2016).

Upon recruitment to the subendothelial space, monocytes differentiate into macrophages. Aside from monocyte influx, the accumulation of macrophages is driven in large part by the proliferation of pre-existing tissue-residing macrophages, which occurs mainly in advanced atherosclerotic lesions (Robbins et al., 2013). These macrophages take up modified apoB lipoproteins that are retained in the subendothelial space, of which minimally modified LDL (mmLDL) is the most prominent. This uptake occurs in various ways. On the one hand, through phagocytosis mediated by scavenger receptor A1 (SRA1), Lectin-like Oxidized Low-Density Lipoprotein Receptor-1 (LOX-1) and CD-36 (Moore and Freeman, 2006; Poznyak et al., 2021). On the other hand, native LDL is also internalized by macrophages, albeit to a lesser extent, through micropinocytosis. Once internalized by the macrophages, LDL undergoes degradation within macrophagic lysosomes, with degradation byproducts being stored as droplets in the cytoplasm. Microscopic analysis showed that the accumulation of these droplets give macrophages the characteristic appearance of cholesterol-laden foam cells (Brown and Goldstein, 1983). These foam cells contribute significantly to plaque growth and instability and are a hallmark of the initial fatty streak phase of atherosclerosis.

### 3.3 Persistent hypercholesterolemia overwhelms foam cells, leading to a pro-inflammatory response

The influx and subsequent proliferation of foam cells is regarded as a main driver of fatty streak formation and subsequent plaque growth. Even though monocyte-derived macrophages are well-recognized contributors to foam cell formation in atherosclerosis, scRNA-seq analysis in mouse models of atherosclerosis have indicated a role for vascular smooth muscle cells (VSMCs) that is far from negligible. Stimulated by TGF-β, which is secreted by -amongst others- macrophages, ECs and T-cells, VSMCs express high levels of smooth muscle α-actin and engage in the production of a complex extracellular matrix containing elastin, proteoglycans and collagen (Bobik, 2006; Bentzon et al., 2014). This matrix forms a fibrous cap that surrounds a core of foam cells (Libby, 2000). Interestingly, recent fate-mapping experiments have evidenced that VSMCs within a fibrous cap are derived from proliferation of a single VSMC in the medial layer of the vessel (Misra et al., 2018). The proliferation of VSMCs and the formation of this fibrous cap marks a critical point for the plaque, after which the likelihood of spontaneous regression of the plaque diminishes (Gofman et al., 1950; Bennett et al., 2016). On the other hand, incorporation of VSMC-derived extracellular matrix into the fibrous cap may increase stability, lowering the chances of plaque rupture and subsequent atherothrombotic events (Bennett et al., 2016). As the plaque progresses, VSMCs migrate into the plaque and undergo transdifferentiation into a variety of transcriptionally heterogenic phenotypes as evidenced by several murine scRNA-seq studies (Hutton et al., 2023). Remarkably, a large subset of these VSMCs gain expression of macrophage markers and engage in the uptake of lipoproteins to become the majority of plaque foam cells, over macrophages (Francis, 2023). scRNA-seq studies in human plaques have confirmed that VSMCs in humans form a heterogeneous population as well (Depuydt et al., 2020), but the extent to which phenotypic switching plays a role in human disease remains a matter of ongoing investigation.

Following periods of persistent hypercholesterolemia, sustained influx of lipoproteins can outpace the metabolic capacities of foam cells. When this happens, pro-inflammatory responses are triggered, such as the NF-κB-dependent secretion of cytokines like tumor necrosis factor α (TNF-α), IL-6 and IL-1β (Figure 2) (Yvan-Charvet et al., 2008). This ultimately leads to the infiltration and activation of pro-atherogenic leukocytes, coupled with amplified lipoprotein modification, and aggravated foam cell formation (Williams et al., 2019). Moreover, cholesterol is incorporated into the cell membrane of foam cells, amplifying inflammatory signaling (Tall and Yvan-Charvet, 2015). Endoplasmatic reticulum stress caused by prolonged lipid overload may trigger foam cells to undergo apoptosis or necrosis, after which they are removed by macrophages in a process called efferocytosis (Neels et al., 2023). Initially beneficial to plaque regression, foam cell death and subsequent clearance of cell debris by efferocytosis diminishes the number of cells present in the plaque. Over time however, the ability of efferocytes to efficiently clear apoptotic and necrotic cells diminishes, resulting in accumulation of cell debris and necrotic core formation. This induces increased plaque vulnerability (Gonzalez and Trigatti, 2017; Neels et al., 2023). In this context, it has been shown that efferocytes release their pro-inflammatory cellular and lipid contents, further contributing to leukocyte recruitment (Kojima et al., 2017).

### 3.4 Inflammasome-mediated inflammation in atherosclerotic plaque formation

As the CANTOS and COLCOT trials generated promising evidence for the anti-atherogenic effects of IL-1β antibodies and colchicine, the inflammasome has amassed attention as the major driving factor of IL-1β-driven inflammation in atherosclerosis. Inflammasomes are located within the cytoplasm of immune cells of the innate immune system. They function as intracellular sensors that respond to damage-associated molecular patterns (DAMPs; released during cellular stress), as well as pathogen-associated molecular patterns (PAMPs; associated with microbes) (Kelley et al., 2019). These distinct patterns are detected by specialized receptors referred to as pattern recognition receptors (PRRs), which include TLRs and nucleotide-binding oligomerization domain-like receptors (NLRs) (Kelley et al., 2019). These NLRs, the most prominent of which include NLR Family Pyrin Domain Containing 1 (NLRP1), NLRP3, and NLR Family CARD Domain Containing 4 (NLRC4), guide the assembly of inflammasomes upon recognition of DAMPs or PAMPs, ultimately leading to activation of caspase-1. This enzyme then converts the inactive forms of the pro-inflammatory cytokines IL-1β and IL-18 into their active counterparts (Kelley et al., 2019). Within atherosclerotic plaques, the accumulation of modified lipoproteins leads to the creation of cholesterol microcrystals that activate the inflammasome through enhanced signaling of the nuclear receptor subfamily three group C member 2 (NR3C2) (Chen et al., 2023). One experimental study definitively established the inflammasome as a link between cholesterol and atherosclerosis, as peritoneal exposure of atherosclerosis-prone mice to these cholesterol crystals was found to be able to trigger inflammation and atherosclerosis in a NLRP3 inflammasome-dependent manner (Duewell et al., 2010). It is therefore unsurprising that genes associated with the NLRP3 inflammasome exhibit substantially higher expression levels in atherosclerotic plaques compared to non-atherosclerotic areas in human arteries (Paramel et al., 2016). These changes were found to be particularly pronounced in patients with symptomatic lesions in a sub-group analysis of the same experiment (Paramel et al., 2016), and an elevated expression of NLRP3 in the aorta was found to correlate with an increased risk of developing coronary artery disease (Zheng et al., 2013). Experimental lentiviral NLRP3 silencing reduced atherosclerotic plaque area, macrophage count within lesions, lipid accumulation, and heightened plaque stability via increased collagen content in *Apoe* ^*−/−*^ mice (Zheng et al., 2014).

The NLRP3 inflammasome promotes inflammation by facilitating the release of IL-1β and IL-18 (Kelley et al., 2019). Of these, IL-1β has been found to play a significant role in promoting endothelial activation by enhancing the expression of endothelial adhesion molecules such as ICAM-1 and VCAM-1 and monocyte adhesion to ECs (Cejkova et al., 2019). Additionally, NLRP3 inflammasome activation induces the release of other key pro-inflammatory cytokines like CCL2, CXCL2, -3 and 8, IL-6 and matrix metalloproteinases (MMPs), which are enzymes that promote fibrous cap dissolution. Therefore, plaque instability is promoted through an increased risk cap rupture and subsequent thrombus formation (Popa-Fotea et al., 2023). These events also contribute to the influx of leukocytes and the uptake of LDL from the intravascular space, thereby perpetuating the cycle of inflammation (Botts et al., 2021). Interestingly, it has been shown that the phenotypic switch and transdifferentiation of VSMCs towards macrophage-like cells is likely dependent on the activation of the NLRP3 inflammasome in VSMCs (Burger et al., 2021). Corroborating the effectivity of mitigating IL-1β activity in human atherosclerosis, depleting IL-1β genetically in *Apoe* ^*−/−*^ mice has been shown to notably reduce atherosclerosis progression (Kirii et al., 2003). IL-18 similarly holds significance as a driver of inflammation and plaque progression. Similarly to IL-1β, it also increases the expression of adhesion molecules and inflammatory cytokines through NF-κB and MAPK signaling, albeit to a lesser extent (Yasuda et al., 2019). IL-18 acts as an important costimulatory cytokine, essential for the production and secretion of interferon-γ (IFN-γ) from T-helper 1 (Th1) cells and NK cells, macrophages, DCs, and VSMCs (Yasuda et al., 2019). Indeed, experimental genetic depletion and overstimulation of IL-18 in *Apoe* ^*−/−*^ mice has shown that the cytokine consistently demonstrates a direct association with the progression of atherosclerotic lesions, operating through an IFN-γ-dependent mechanism (Whitman et al., 2002; Elhage et al., 2003; Tan et al., 2010).

### 3.5 Macrophages: beyond the M1-M2 paradigm

Not all macrophages within the plaque exhibit identical pro-inflammatory characteristics. Macrophages have the capacity to undergo polarization, dictated by their microenvironment, leading to phenotypic and functional changes (Wu et al., 2023). Traditionally, macrophage phenotypes have been categorized into two groups: pro-inflammatory M1 and anti-inflammatory M2 macrophages (Moore et al., 2013). The initial categorization was based on their specific *in vitro* stimulation factors. Subsequent research, both *in vitro* and in murine atherosclerosis models, have led to one overarching principle: M1 macrophages are linked to the promotion of plaque inflammation, whereas M2 macrophages are connected to the resolution of plaque inflammation (Zhao et al., 2023). In these studies, M1 macrophages have been detected within atherosclerotic plaques in humans, specifically localized in lipid-enriched regions spatially separate from M2 macrophages (Stöger et al., 2012). Their accumulation and subsequent apoptosis or necrosis leads to expansion of the necrotic core, which causes plaque progression and destabilization. Aside from the pro-inflammatory cytokines TNF-α and IL-1β, M1 macrophages produce MMPs, which, as discussed, dissolve the fibrous cap and promote plaque instability. They also secrete high levels of IL-6 and IL-12, which promote differentiation of naïve T cells into pro-inflammatory Th1 cells (Mosser, 2003). On the contrary, murine atherosclerosis models have suggested that M2 macrophages promote plaque regression (Feig et al., 2012). M2 macrophages secrete high levels of IL-10, which promotes differentiation of naïve T cells into anti-inflammatory Th2 cells. Furthermore, IL-10 promotes plaque stabilization through extracellular matrix formation. Notably, consistent findings from murine models indicate a reduction in macrophage population, at times accompanied by an increased presence of M2 macrophages, correlates with plaque regression (Jinnouchi et al., 2020). Although M2 macrophages have also been identified within human plaques, uncertainty remains regarding their role in plaque development (Stöger et al., 2012). Nevertheless, it’s imperative to acknowledge that the translatability of these observations to humans is in some ways limited due to the inherent differences in macrophage subtypes between mice and humans. For instance, while general functional characteristics of macrophage subsets, including the factors that steer their differentiation, show a high degree of conservation between mice and humans, surface markers seem to differ substantially between the species (Chinetti-Gbaguidi et al., 2015).

Today, the significance of the M1-M2 paradigm in atherosclerosis is a major area of debate. scRNA-seq has allowed investigators to identify previously undiscovered macrophage subtypes characterized by distinct gene expression profiles involved in atherosclerosis over recent years. Importantly, these subtypes do not necessarily align with the two subtypes defined by the classical M1-M2 paradigm. Many of these scRNA-seq datasets are publicly available, a meta-analysis of which was recently carried out by Zernecke et al. (2023). They found that in murine models of atherosclerosis, as many as 10 functionally distinct macrophage subpopulations could robustly be identified. These cell clusters appeared to be conserved in human atherosclerosis. To date, efforts to further characterize macrophage subpopulations are increasing. These have recently been reviewed elsewhere (Wieland et al., 2024).

### 3.6 Neutrophils attract monocytes to the vessel wall and modulate macrophage phenotypes in atherosclerosis

Neutrophils have long been overlooked in atherosclerosis research, possibly due to their short lifespans and phenotypic plasticity, making their *in vivo* detection challenging (Zhang et al., 2023). In recent years, however, experimental findings have shed a new light on these cells in the context of atherosclerosis. Neutrophils are recruited to the activated endothelium by chemokines such as CCL-1 and CXCR2 (Drechsler et al., 2010; Lam et al., 2018). Similarly to monocytes, neutrophils bind to activated ECs in a P- and E-selectin and CCR2-dependent manner (Lam et al., 2018; Zhang et al., 2023). Upon adhering to the endothelium, neutrophils produce reactive oxygen species (ROS), thereby contributing to the oxidation of lipoproteins within the endothelium and the permeability of the vessel wall (Domínguez-Luis et al., 2019; Lian et al., 2019). Neutrophils further increase EC permeability and facilitate the transmigration of both neutrophils and other immune cells through secretion of pro-inflammatory cytokines TNF-α and IL-1β (DiStasi and Ley, 2009; Zhang et al., 2023).

In a landmark study of murine atherosclerosis, researchers examined the aortas of neutropenic mice and their high-fat diet-fed control counterparts. In the aortas of the neutropenic mice, the number of monocytes and macrophages was significantly reduced, as well as the size of atherosclerotic lesions. These findings suggest that neutrophils play a role in the accumulation of monocytes and monocyte-derived macrophages within atherosclerotic lesions (Drechsler et al., 2010). Recent findings from a murine model of advanced atherosclerosis indicate that the pro-atherosclerotic activity of neutrophils depends on signaling by Signal transducer and activator of transcription 4 (STAT4) (Keeter et al., 2023). Several mechanisms have been implicated in the link between neutrophils and atherogenesis. For instance, a recent study used intravital microscopy in *Apoe* ^*−/−*^ mice to demonstrate that when stimulated by activated ECs, neutrophils release neutrophil extracellular traps (NETs) along the arterial wall, consisting mostly of DNA strands, histones and neutrophil granules. This release promoted monocyte adhesion independently of receptor signaling (Schumski et al., 2021). Another study demonstrated that exposure to cholesterol crystals could induce the release of NETs from neutrophils in both a mouse model of atherosclerosis where it induced the release of pro-inflammatory cytokines from macrophages (Warnatsch et al., 2015) and in human neutrophils (Awasthi et al., 2016). *In vitro* studies with human macrophages have demonstrated that citrullinated histones, associated with NETosis, enhance the oxidation of LDL and the formation of foam cells (Haritha et al., 2020). Finally, it has been shown that DNA and neutrophilic granules, such as those present in NETs, facilitate the growth of atherosclerotic plaque in mice in a manner dependent on an increased production of interferon-α.

Furthermore, neutrophils enhance monocyte chemotaxis and adherence by releasing CCL2 and pentraxin 3 (Winter et al., 2018; Popa-Fotea et al., 2023), demonstrating that neutrophils are able to initiate efficient monocyte extravasation into the subendothelial space. Beyond their role in monocyte recruitment, neutrophils have also been implicated in modulating macrophage phenotypes. Within the plaque, neutrophils secrete azurocidin and α-defensins, which induce a shift in macrophages towards a T-helper cell (Th)-17 stimulating M1 phenotype through β2-integrin signaling and subsequent interferon-γ release (Zhang et al., 2023). Furthermore, experimental exposure of lipid crystals to neutrophils has been shown to lead to increased NET release. This in turn triggered M1 polarization in *Apoe* ^*−/−*^ mice (Warnatsch et al., 2015). Moreover, the effect of neutrophils on plaque content and macrophage functionality was evidenced in a study, in which *in vitro* incubation of macrophages with neutrophil-derived defensin increased the expression of CD36, which in turn enhanced the uptake of LDL and promoted foam cell formation (Quinn et al., 2011). These results were corroborated in a recent study, in which human neutrophils exposed to LDL were visualized using fluorescent microscopy. In this study, LDL induced NET release *in vitro*, which in turn promoted LDL oxidation, LDL accumulation and foam cell formation (Haritha et al., 2020). Finally, a recent study has established a role for NET-derived histone H4 in exacerbating plaque instability. Here, histone H4 exerted a deleterious function on the cell membrane of VSMCs, ultimately contributing to an increase in plaque instability in mice and humans (Silvestre-Roig et al., 2019). These findings underline the important role neutrophils play in promoting atherogenesis and plaque inflammation.

## 4 The role of the adaptive immune system in atherosclerosis

### 4.1 Auto-antigen-specific T cells modulate atherosclerosis

T cells express the T-cell receptor (TCR) in conjunction with co-receptors that align with their specific function and T cell subtype. CD4 co-receptor-expressing cells differentiate into Th cells following antigen presentation and are the most widely studied T cell subtype in the context of atherosclerosis (Popa-Fotea et al., 2023). Expression of the CD8 co-receptor, on the other hand, is found in naïve and effector cytotoxic T cells (Popa-Fotea et al., 2023). These cells regulate immune responses to antigens presented by antigen-presenting cells (APCs) such as macrophages and DCs on their major histocompatibility complex (MHC) class II, as well as by all nucleated cells on MHC class I. Concurrent interactions between TCRs and antigens on MHC, coupled with stimulation by co-stimulatory molecules presented on APCs, drive the clonal proliferation of T cells and determine their phenotype (Saigusa et al., 2020).

During the early stages of atherogenesis, T lymphocytes are abundantly present (Saigusa et al., 2020). They are drawn to the activated endothelium by a variety of chemokines, including CCL5, CXCR10, and CXCL16 (Wuttge et al., 2004; Heller et al., 2006; Li et al., 2016). Subsequently, these T cells migrate into the endothelium through the interaction of P-selectin and PSGL-1 *in vivo* (MacRitchie et al., 2019). In *Apoe* ^*−/−*^ mice, a considerable portion of plaque-resident CD4^+^ T cells exhibit CD44 expression, a T cell activation marker that allows discriminating effector and memory T cells from naïve T cells. This suggests that these lymphocytes have previously encountered and been activated by their corresponding antigen (Koltsova et al., 2012). In recent years, omics approaches have been of great value in exploring this hypothesis. Mass cytometry in human atherosclerotic plaques confirmed that chronically activated and differentiated T cell phenotypes predominated over the naïve population (Fernandez et al., 2019). This finding was corroborated by two separate studies that used single-cell RNA and TCR sequencing to establish that many T cells found in human coronary plaques were antigen-experienced memory cells that had clonally expanded within the plaque (Chowdhury et al., 2022; Depuydt et al., 2023). Comparison of T cell subsets in the plaque and peripheral blood revealed that it is mainly effector CD4^+^ T cells, and not CD8^+^ T cells that undergo clonal expansion within the plaque (Depuydt et al., 2023).

Regarding the role these antigen-experienced T cells play in atherogenesis, the depletion of CD44 in a murine model of myocardial infarction resulted in heightened inflammatory leukocyte infiltration and increased cytokine expression (Huebener et al., 2008). Identifying the antigen specificity of T cells *in vivo* has proven technically difficult. ApoB has been identified as an auto-antigen that likely is important in this regard. Immunization with human ApoB reduced atherosclerosis significantly in *Apoe* ^*−/−*^ mice (Fredrikson et al., 2003; Chyu et al., 2022). Additionally, both in murine atherosclerosis models and humans with cardiovascular disease, a population of apoB-specific T-helper cells was identified in experiments that used recombinant MHC (Kimura et al., 2018). Other auto-antigens that have been implicated in atherogenesis include beta2-glycoprotein I (Profumo et al., 2010), cathelicidin (Mihailovic et al., 2017) and collagens (Lio et al., 2020). These findings underscore the important role that antigen-specific T cells assume in modulating inflammation in cardiovascular disease. The diverse range of potential immune-dominant antigens implicated in atherogenesis may help clarify the complex and sometimes contradictory roles of T cells in atherosclerosis, which could vary depending on the disease stage and the specific antigens involved.

### 4.2 CD4^+^ T-helper cells: different subtypes have different functions in atherosclerosis

**FIGURE 3:**
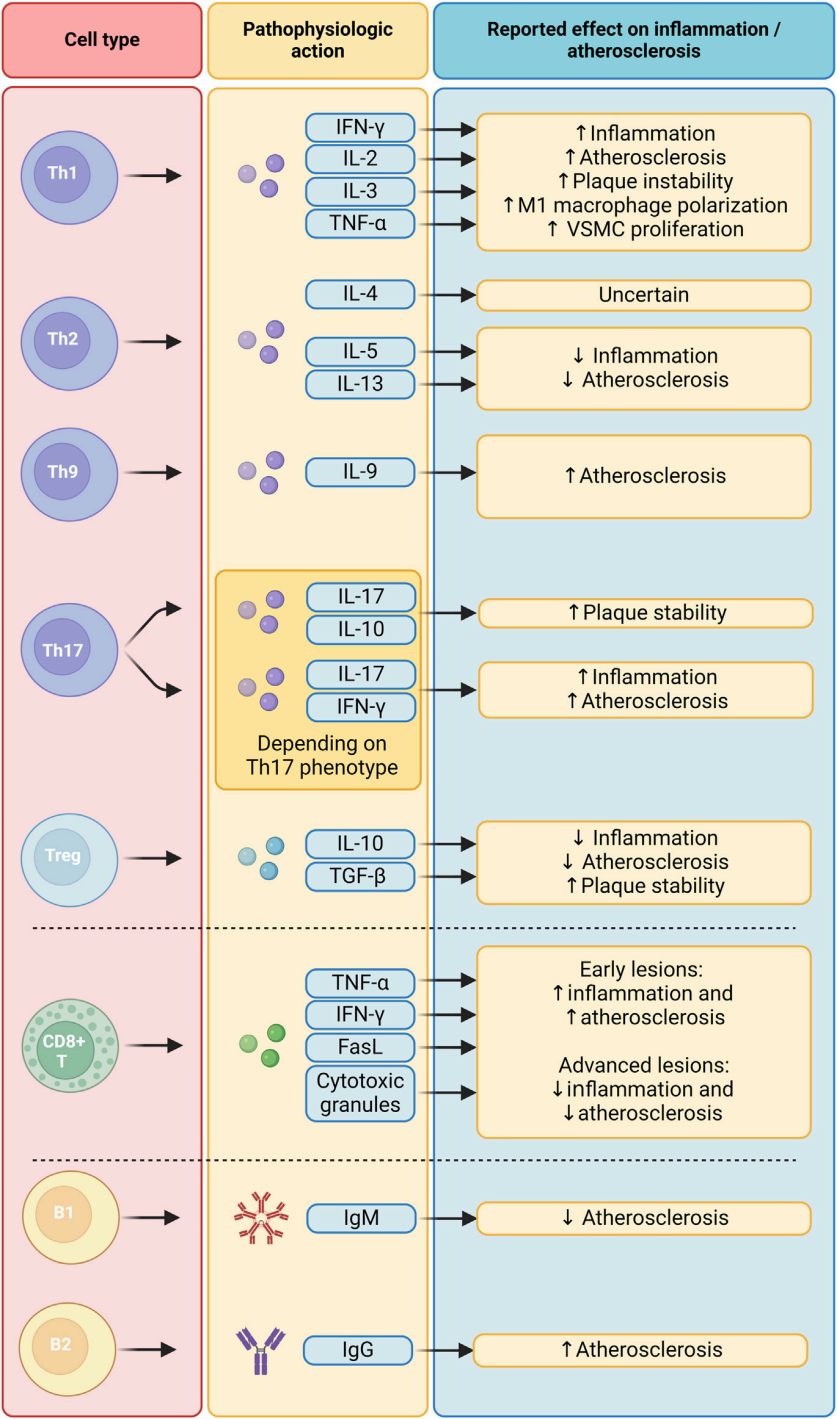
Functions of the adaptive immune system in atherosclerosis. B1, B1 cells; B2, B2 cells; CD8^+^ T, CD8^+^ T cells; FasL, Fas Ligand; IFN-γ, Interferon gamma; IgG, Immunoglobulin G; IgM, Immunoglobulin M; IL-10, Interleukin 10; IL-13, Interleukin 13; IL-17, Interleukin 17; IL-2, Interleukin 2; IL-3, Interleukin 3; IL-4, Interleukin 4; IL-5, Interleukin 5; TGF-β, Transforming Growth Factor beta; Th1, T helper 1 cells; Th17, T helper 17 cells; Th2, T helper 2 cells; Th9, T helper 9 cells; TNF-α, Tumor Necrosis Factor alpha; Treg, Regulatory T cells; VSMC, Vascular Smooth Muscle Cell.

T-helper cells, which are classified into subtypes based on their signature cytokines and transcription factors, play a variety of roles in the context of atherosclerosis (Figure 3). Th1 cells, which express the transcription factor T-bet as a lineage-defining marker, predominate in number among all T cell subtypes in the plaque (Li et al., 2016; Wolf and Ley, 2019). Their role is pro-inflammatory and pro-atherogenic due to the secretion of IFN-γ, IL-2, IL-3, and TNF-α (Popa-Fotea et al., 2023). This is highlighted by genetic depletion experiments of T-bet and IFN-γ, which have been found to inhibit plaque progression and instability in *Ldlr* ^*−/−*^ mice (Buono et al., 2003; Buono et al., 2005). Furthermore, IFN-γ plays a role in plaque progression by promoting LDL oxidation and uptake of modified LDL by foam cells, polarizing macrophages to their M1 subtype, and promoting the proliferation of VSMCs (Rocha et al., 2008; Lee et al., 2021).

On the other hand, the precise role of Th2 cells, which mainly produce IL-4, IL-5, and IL-13, remains a topic of debate (Winkels et al., 2018). Levels of Th2 cells and IL-4 released in peripheral blood are inversely correlated with carotid intima-media thickness in a healthy study population, even after correcting for other cardiovascular disease risk factors (Engelbertsen et al., 2013). However, depletion of IL-4 has shown conflicting effects on plaque growth in mice (King et al., 2002; Mallat et al., 2009). IL-5 and IL-13, on the other hand, have definite anti-inflammatory and anti-atherogenic effects (Saigusa et al., 2020).

The role of Th9 cells in atherosclerosis, too, remains uncertain, but preliminary evidence points to a pro-atherogenic function. The main cytokine produced by this subset is IL-9. Several clinical studies found that IL-9 levels, but not the number of Th9 cells, are higher in patients with atherosclerotic disease (Gregersen et al., 2013; Lin et al., 2013). In murine models of atherosclerosis, IL-9 has been found to have pro-atherogenic effects (Zhang et al., 2015). More research is needed to fully elucidate the role of Th9 cells and IL-9 in the pathogenesis of atherosclerosis.

Th17 cells feature the expression of transcription factor RORγt as their lineage-defining factor. Previous murine *in vitro* studies have elucidated the complex nature of Th17 cells: due to their high level of plasticity, their phenotype (pro-inflammatory or anti-inflammatory) is highly dependent on the cytokines they are polarized with (McGeachy et al., 2007; Ghoreschi et al., 2010; Lee et al., 2012). This heterogenic nature of Th17 cells might be the reason why previous research efforts have found conflicting roles for this cell type in atherosclerosis. Indeed, Th17 cell counts correlated with atherosclerotic plaque size in *Apoe* ^−/−^ mice. Neutralizing antibodies against IL-17 could diminish plaque size and leukocyte infiltration (Nordlohne et al., 2018). On the other hand, one study found increased plaque stability in *Ldlr* ^*−/−*^ mice with artificially increased Th17 cell counts (Brauner et al., 2018).

T follicular helper (Tfh) cells are a specialized subset of CD4^+^ T cells, hallmarked by the expression of B-cell lymphoma 6 (Bcl-6), that play a critical role in the formation and maintenance of germinal centers, where they aid B cells in producing high-affinity antibodies. They facilitate B cell differentiation and antibody class switching through the secretion of cytokines and direct cell-cell interactions (Qi et al., 2023). Evidence from experimental studies point to divergent functions of Tfh cells in atherosclerosis. An experimental study found that Tfh cells isolated from *Apoe* ^−/−^ mice had a gene expression profile that was more pro-inflammatory than those isolated from wild-type mice. This effect was mediated by enhanced IL-27 production from dendritic cells (Ryu et al., 2018). Another study showed that marginal zone B cells inhibit pro-atherogenic Tfh cell activity in *Ldlr* ^−/−^ mice (Nus et al., 2017). Additionally, genetic depletion of Tfh cells in *Apoe* ^−/−^ mice led to a reduction of atherosclerosis (Gaddis et al., 2018). In a recent experiment in an atherosclerotic mouse model, however, genetic depletion of Tfh cells led to an aberrant antibody response of marginal zone B cells and an associated increase in atherosclerotic plaque formation (Harrison et al., 2024). These conflicting findings might reflect the recently uncovered heterogeneity of Tfh subsets (Seth and Craft, 2019), warranting further delineation of the roles of these subsets in atherosclerosis.

Regulatory T cells (Tregs) express the forkhead box P3 (FoxP3) and have been identified as having anti-inflammatory and atheroprotective effects. Depletion of this population has been found to promote atherosclerosis in *Ldlr* ^*−/−*^ mice (Klingenberg et al., 2013), and a strong negative correlation between Treg cells and atherosclerosis exists in humans (George et al., 2012). Tregs exert their anti-inflammatory function through the secretion of IL-10 and TGF-β (Saigusa et al., 2020). Depletion of either these factors was found to increase atherosclerotic plaque size and instability in murine models (Pinderski et al., 1999; Mallat et al., 2001). The population of apoB-specific Tregs appears to be diminished in the peripheral blood of patients with cardiovascular disease compared to healthy controls (Pinderski et al., 1999). Interestingly, as atherosclerosis progresses, Treg numbers in peripheral blood and the plaque diminishes in favor of effector Th1/Th17 cells in both murine models and in^*-*^ mice and humans. A substantial proportion of Tregs that remain were found to have acquired Th1 and Th17-defining transcription factors while simultaneously losing the expression of FoxP3 (Butcher et al., 2016; Wolf et al., 2020). These findings suggest that a decline in the number of bona fide Treg cells, along with their increasingly pro-inflammatory phenotype, might be an independent driver of disease progression in atherosclerosis.

### 4.3 The role of CD8^+^ T cells in plaque inflammation remains to be elucidated

CD8^+^ T cells activate and differentiate into effector T cells following the interaction of their TCR with an antigen presented on MHC class I molecules. They then undergo clonal expansion and produce TNF-α, IFN-γ, Fas-ligand, and cytotoxic granules. This leads to the induction of apoptosis or necrosis in the targeted cell (Schäfer and Zernecke, 2020). While CD4^+^ T cell functions have been extensively studied in atherosclerosis, the role of CD8^+^ cytotoxic T cells in this context is less well known (Figure 3).

Similarly to CD4^+^ T cells, CD8^+^ T cells are prominently present within atherosclerotic plaques in both mice and humans (Cochain et al., 2018). The precise contribution of CD8^+^ T cells to atherosclerosis, however, needs to be studied further. One piece of evidence for their mechanistic role in atherosclerosis was provided by an experiment involving the depletion of CD8^+^ T cells in *Apoe* ^−/−^ mice using anti-CD8α antibodies, which resulted in a significant reduction in plasma CCL2 levels, as well as the accumulation of macrophages and a reduction in atherosclerotic plaque size in the early stages of disease progression. These effects could be mediated by their cytotoxic effects and subsequent growth of the necrotic plaque core (Kyaw et al., 2013). A recent study which employed depletion of the CD8^+^ T cell line in a murine model of atherosclerosis, found that CD8^+^ T cells induce VSMC dedifferentiation toward a phenotype associated with plaque calcification (Schäfer et al., 2024). On the other hand, an experiment with a longer follow-up time contradicted these findings and provided evidence for a potential atheroprotective role of CD8^+^ T cells in advanced lesions (van Duijn et al., 2019). Another study showed that in *Apoe* ^*−/−*^ mice, a subset of regulatory CD8^+^ T cells is involved in the regulation of Tfh cell activity, thereby reducing atherosclerosis (Clement et al., 2015). These conflicting results illustrate the complexity of CD8^+^ T cell functions in atherosclerosis and suggest that their impact may vary depending on the stage of the disease. Given these discrepancies, further investigation into the precise role of CD8^+^ T cells is warranted to better comprehend their influence on atherogenesis.

### 4.4 B1 and B2 cells appear to have opposing roles in atherosclerosis

The presence of B cells in atherosclerotic plaques has been confirmed in scRNA-seq studies, but it appears they are generally sparse (Winkels et al., 2018; Fernandez et al., 2019). Instead, they predominantly inhabit the lymphoid tissue surrounding the arterial wall and the peritoneal cavity (Mangge et al., 2020). These cells are traditionally categorized into two distinct subtypes (Figure 3). B1 cells, integral to the innate immune response, secrete germ-line encoded IgM antibodies of low affinity aimed at common pathogens. Conversely, B2 cells necessitate stimulation from T-follicular helper cells (Tfh) to mature into plasma cells within germinal centers, ultimately releasing high-affinity IgG antibodies. In the context of atherosclerosis, B2 cells localize to and interact with T cells and APCs in unique adventitial structures termed artery tertiary lymphoid organs (Mohanta et al., 2014).

Observational studies reveal a dichotomous impact of these subgroups. Titers of IgM antibodies targeting apoB exhibited an inverse correlation with atherosclerosis, while titers of apoB-specific IgG antibodies displayed a positive correlation with disease progression in both murine models and humans (Karvonen et al., 2003; Tsimikas et al., 2007; Bjö et al., 2016). Intriguingly, the depletion of B1 cells through splenectomy in atherosclerosis-prone mice exacerbates the formation of atherosclerotic lesions, whereas artificial expansion of B1 cells attenuates atherosclerosis (Kyaw et al., 2011; Srikakulapu et al., 2017; Hosseini et al., 2018). This effect is hypothesized to result from the adverse effects of apoB-specific IgM antibodies on macrophage-mediated lipoprotein uptake (Kyaw et al., 2011). Conversely, an initial experiment involving the broad antibody-mediated removal of all B2 cells in mouse models of atherosclerosis has shown to mitigate inflammation and atherosclerosis (Ait-Oufella et al., 2010). However, the precise extent and characterization of B cells’ involvement in human atherosclerosis remain subjects for further investigation. Again, it is important to consider the antigen-specificity of B cells involved in atherogenesis, as this may significantly impact the role of B cells in this disease.

## 5 Conclusion

In conclusion, this review describes atherosclerosis as a disease of low-grade vascular inflammation that is driven by a myriad of different pro-inflammatory processes and cellular players. A large body of evidence in this regard is derived from experimental murine models of atherosclerosis for obvious reasons: it is impossible to capture the complexity of this disease relying solely on *in vitro* experiments. Nevertheless, it is important to note that significant differences exist between the cardiovascular systems of mice and humans that should be considered when translating findings. For instance, shear stress levels are higher in the murine vasculature (Greve et al., 2006). Furthermore, spontaneous plaque rupture is rare in mice, necessitating the use of ligation as a model of plaque rupture (Schwartz et al., 2007). With regards to the murine and human immune system, important differences exist as well. Many cytokines lack a cross-species counterpart (Shay et al., 2013) and the distribution of peripheral leukocytes is different between the two species (Mestas and Hughes, 2004). Consequently, an increasing number of experiments are attempting to more closely replicate the conditions of the human vasculature by employing vasculature-on-a-chip systems or organoids (Abaci and Esch, 2024). However, despite the limitations of mouse models, they continue to play a vital role in research in this field, offering the advantages of a fully developed mammalian immune and cardiovascular system.

The success of several clinical outcome trials has cemented the role of anti-inflammatory therapeutic strategies as means to put plaque inflammation to a halt, thereby reducing the ASCVD event rate in patients with residual inflammatory risk. However, rather than broadly targeting inflammation, which has been shown to lead to an increase in infection rate due to systemic immune suppression, the key to successfully combating inflammation in patients that are at risk of ASCVD in the future will lie in zooming in on more specific pro- and anti-inflammatory processes that are crucial to disease progression and targetable in humans. One example currently under investigation is the use of rituximab in patients who experienced a myocardial infarction in order to alter B cell populations (Zhao et al., 2022a). Another avenue that is currently being explored is the use of low-dose aldesleukin, a recombinant IL-2, in patients with stable ischemic heart disease to specifically stimulate Tregs (Zhao et al., 2022b). Luckily, the toolbox of atherosclerosis research has been enriched over recent years has facilitated this, with high-throughput methodologies such as scRNA-seq as an important means of exploring the innate and adaptive immune system in atherogenesis in more detail.

This review has provided an overview centered around the knowns and the unknowns of the cellular players in atherosclerotic inflammation, thereby providing a basis on which future research efforts to characterize the plaque immune landscape can build.
